# Mechanical control of the insect extracellular matrix nanostructure

**DOI:** 10.1126/sciadv.adw5022

**Published:** 2026-01-02

**Authors:** Yuki Itakura, Housei Wada, Sachi Inagaki, Shigeo Hayashi

**Affiliations:** ^1^ Laboratory for Morphogenetic Signaling, RIKEN Center for Biosystems Dynamics Research, Kobe 650-0047, Japan.; ^2^ Department of Biology, Kobe University Graduate School of Science, Kobe 657-8051, Japan.

## Abstract

Nanoscale modifications of apical extracellular matrix (ECM) have created various functional surfaces with distinct physical properties, exemplified by structural coloration and superhydrophobicity in animals and plants. To reveal the mechanisms, we investigated cuticle morphogenesis in *Drosophila* olfactory organs, where hundreds of ~50-nanometer nanopores in the cuticle covering the olfactory neurons permit selective odorant entry. We showed that zona pellucida domain (ZPD) proteins form the cell type–specific ECM compartments before cuticle secretion, and its disruption leads to less and irregularly sized nanopores. The ZPD protein Dusky-like controls the formation of the outermost layer of the cuticle, the envelope. Trynity, Nyobe, Neo, and Morpheyus form matrices with specific mixing and sorting properties, termed “cloud ECM,” which restrict cell growth and movement. This work identifies a previously unidentified role for ZPD proteins as modular units that establish the mechanical environment essential for nanoscale ECM morphogenesis, opening a previously unexplored context for these biomimetically important structures.

## Teaser

Zona pellucida domain proteins form elastic ECM, controlling the cuticular nanopore formation in the insect olfactory organ.

## INTRODUCTION

The outer surface of living organisms is coated with various forms of apical extracellular matrices (aECMs), playing roles in protective functions and mechanical support. aECM is often modified to perform other specialized functions as well, such as selective light reflection, water repellency, molecular filtering in sensory organs, and surface ornamentation (*1*–*3*). Structures of functional aECMs have been widely adopted in the biomimetic fabrication of inorganic materials for industrial uses (*4*, *5*). A better understanding and control of nanoscale structures in biocompatible organic materials would thus greatly expand the potential for biomimetic research and applications (*6*, *7*). However, research in this direction has been impeded by the lack of understanding of the fundamental biological mechanisms underlying ECM nanopatterning.

Cuticle, the aECM in insects, is a multilayered structure comprising the envelope, epicuticle, and chitin-rich procuticle, which form in an outside-in order (*8*, *9*). It has been used as the substrate for extensive evolutionary functionalization of diverse physical mechanisms, such as structural coloration and sensory functions. Wigglesworth (*8*) suggested the crystallization of cuticular substances as a structural basis for iridescent coloration. However, the role of self-organization in ECM nanopatterning has not been addressed because of a lack of information about the molecules acting in the early developmental stage of the epidermis with specific cuticle functions.

**Fig. 1.**
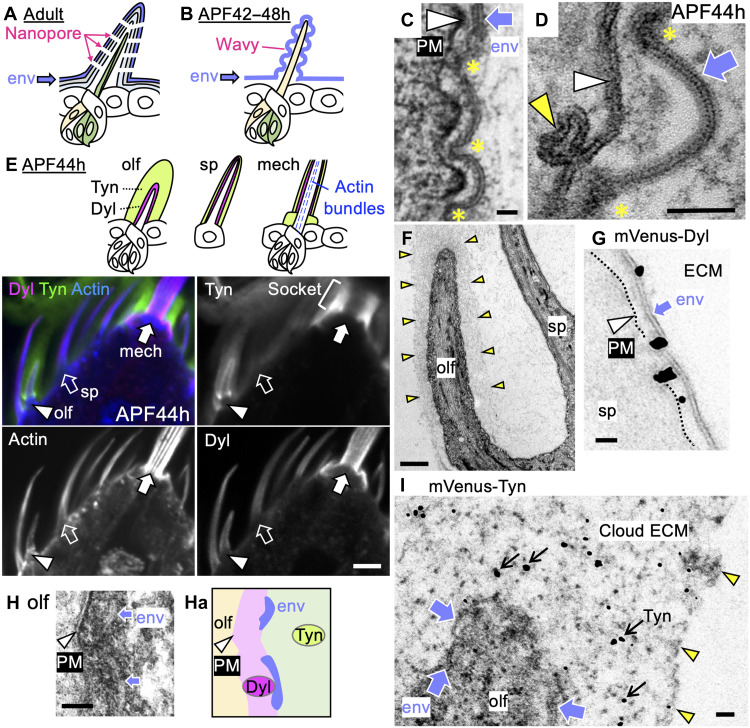
Layered ECM organization of the olfactory hair cell. (**A**) A diagram of an adult olfactory bristle. The envelope (env) is the outermost layer of the cuticle. The nanopores allow odor molecules to reach the dendrites of sensory neurons (green). (**B**) Olfactory hair cell at 42 to 48 hours after puparium formation (APF). The wavy env is formed above the plasma membrane (PM) of the hair cell (yellow). After cuticle formation, the hair cell retracts and is replaced by the dendrite. APF42–48h, 42 to 48 hours AFP. (**C**) An electron micrograph of a wavy env (arrow) over the wavy PM (arrowhead). The innermost (yellow asterisks) and outermost parts of curved env and PM are placed in synchrony. (**D**) The ladder-like env structure (arrow) with electron-dense innermost parts (yellow asterisk) and the lipid bilayer of PM (white arrowhead) are shown. The yellow arrowhead indicates a membrane invagination often observed close to the innermost part of the env. (**E**) Top: The diagram of olfactory hairs (olf), spinules (sp), and mechanosensory bristles (mech) with localization patterns of Dusky-like (Dyl), Trynity (Tyn), and filamentous actin (F-actin). Bottom: Fluorescence images of endogenous mScarlet::Dyl, mVenus::Tyn, and F-actin staining. (**F**) Transmission electron microscopy (TEM) image of olf and sp. The outer boundary of the cloudy material (cloud ECM) around the olf is indicated by yellow arrowheads. (**G**) Immunoelectron microscopy (Immuno-EM) visualization of Dyl (gold-enhanced electron-dense particles) in sp. (**H** and **Ha**) Ruthenium red (RR) staining of olf and the schematic diagram showing the position of PM (arrowheads), Dyl, env, and Tyn. (**I**) Tyn signal (gold-enhanced electron-dense particle; arrow) detected in the cloud ECM. Arrowheads indicate the outer boundary of cloud ECM. Scale bars, 5 μm (E), 1 μm (F), and 50 nm (others).

Studying the nanopores, ~50-nm-diameter cuticular pores with a molecular filtering function, in the olfactory organ of *Drosophila* (Fig. 1A), we previously showed that the first sign of nanoscale structural modification appears at the earliest stage of cuticle formation, with the appearance of the wavy curved envelope (Fig. 1, B and C), in which the bottom of the wave closest to the plasma membrane subsequently forms the nanopore (*10*). *gore-tex/Osiris23* (*gox/Osi23*) (*10*, *11*) regulates this process by introducing the requisite “waviness” to the envelope. Folded or invaginated plasma membranes are frequently associated with prospective pore-forming regions (*10*) (Fig. 1D), leading to the hypothesis that the wavy plasma membrane serves as the template for envelope curvature (*10*). Because Gox/Osi23 protein is present in the endoplasmic reticulum (*12*), its influence on cuticle patterning is necessarily indirect. Further study of aECM materials and their mechanical properties is needed to gain a fuller mechanistic understanding of cuticle nanopatterning.

Zona pellucida domain (ZPD) proteins are conserved apical ECM components with the self-assembly property present in mammalian egg coats, antibacterial filaments in the urinary tract, tectorial membrane in the inner ear, and other biological processes (*13*–*16*). In *Drosophila*, many ZPD proteins are expressed in epidermal tissues before cuticle formation in a cell type–specific manner and set up the cuticle shapes of tracheal tubules and epidermal denticles (*17*–*20*). In nematode, several ZPD proteins and other secreted proteins form transient aECM (precuticle) associated with specific epidermal cell types and control the pattern of collagen-based cuticles (*21*–*24*).

Here, we address the roles of ZPD proteins in cuticle nanopatterning, focusing on Dusky-like (Dyl), Trynity (Tyn), Nyobe (Nyo), and Morpheyus (Mey), all of which have been shown to form compartmentalized aECM and play critical roles in cuticle formation (*17*, *20*, *25*–*29*). These findings align with emerging evidence from both insects and nematodes, suggesting that transient and spatially patterned aECM structures represent a conserved mechanism for shaping epithelial nanoarchitectures (*24*, *30*).

## RESULTS

### Cell type–specific modular organization of ZPD proteins

The envelope layer, the first layer of the cuticle to be formed, emerges above the cell surface as a 20-nm-thick layer that appears ladder-like structure in cross section at ~44 hours after puparium formation (APF; Fig. 1, B to D). We compared the ZPD protein distribution surrounding the olfactory hair (olf) cell of sensilla basiconica in the maxillary palp with that over the spinule (sp), a poreless epidermal protrusion, and the mechanosensory hair (mech), which produces a flat envelope and no nanopores.

Dyl and Tyn were visualized using protein-tagging and immunostaining techniques, and consistent patterns were observed (Fig. 1E and fig. S1A). Dyl formed a tight layer surrounding the surface of sensory hair cells and epidermis, with enhanced signals in sp and mech (Fig. 1E). In contrast, Tyn was prominently enriched in the olf, forming thick layers covering the olf (Fig. 1E). Cloudy extracellular materials were also observed specifically on the olf under transmission electron microscopy (EM; Fig. 1F). We named this structure “Cloud ECM.”

Immunoelectron microscopy (immuno-EM) was performed to detect the localization of mVenus-tagged knock-in alleles of Dyl and Tyn. Because of the high spatial resolution and the sporadic nature of signal detection by gold enhancement, the signal appeared as discrete spots. Dyl was detected mainly between the envelope and cell membrane of sp (Fig. 1G). Ruthenium Red (RR), which stains acidic mucopolysaccharides and glycosaminoglycans enriched in ECM (*31*), stained densely in the membrane-proximal zone and loosely the outside zone on the olf (Fig. 1H). At the border of the two zones, negatively stained convex structures around 50 nm above the plasma membrane, which are likely the envelope, were observed (Fig. 1H). Thus, RR stains Dyl region. Tyn signals were broadly distributed in the cloud ECM (Fig. 1I).

We used *neuralized-gal4* (*neur-gal4*) to knock down Dyl and Tyn expression. *neur-gal4* is widely used as a gene expression driver to target sensory organ precursor cells and their progeny—including neurons, sheath cells, socket cells, and hair cells—particularly during bristle development (*32*, *33*). We confirmed the RNA interference (RNAi) effects on matrix protein levels using mScarlet::Dyl and mVenus::Tyn fluorescence signals (fig. S1B). In Dyl RNAi, Dyl signal on the olf was reduced, whereas in Tyn RNAi, the Tyn ECM on the olf was absent, while the sp and epidermis remained intact. Moreover, loss of Dyl did not alter Tyn distribution, and vice versa. These observations demonstrate the effectiveness and specificity of each RNAi and that the localization of Dyl and Tyn is independent of each other.

Other ZPD proteins, Nyo, Mey, and Neo, were detected on the olf, mech, and sp (fig. S1C). Those proteins occupied distinct territories surrounding the olf (fig. S1, D to F). On the basis of the confocal staining and the immuno-EM results, we tentatively classified aECM of the olf into three layers. Layer I covers immediately above the cell surface and is enriched with Dyl (fig. S1G). At 38 hours APF, Tyn, Neo, and Mey begin to cover the region above layer I (layer II; fig. S1, H to J). Tyn and a part of Mey expand further distally to form layer III (fig. S1, D and F). At 44 hours APF, Nyo and Neo occupy layers II and III, respectively. The cloud ECM corresponds to layers II and III. Components of layers II and III appear to move through layer I into the outer zone, suggesting that layer I aECM permits the diffusion of the precursor components of layers II and III. This order is opposite to the construction of the cuticle, which proceeds from the outside to the inside. In summary, layer I (Dyl) covers all epidermis and its derivatives in 44 hours APF. Olf is specifically covered by cloud ECM (layers II and III). In the period between 38 and 39 hours APF, the olf hair cells grow out from the surface of the maxillary palp into the territory occupied by cloud ECM (fig. S1, J and K).

### ZPD proteins self-organize to build distinct ECMs

**Fig. 2.**
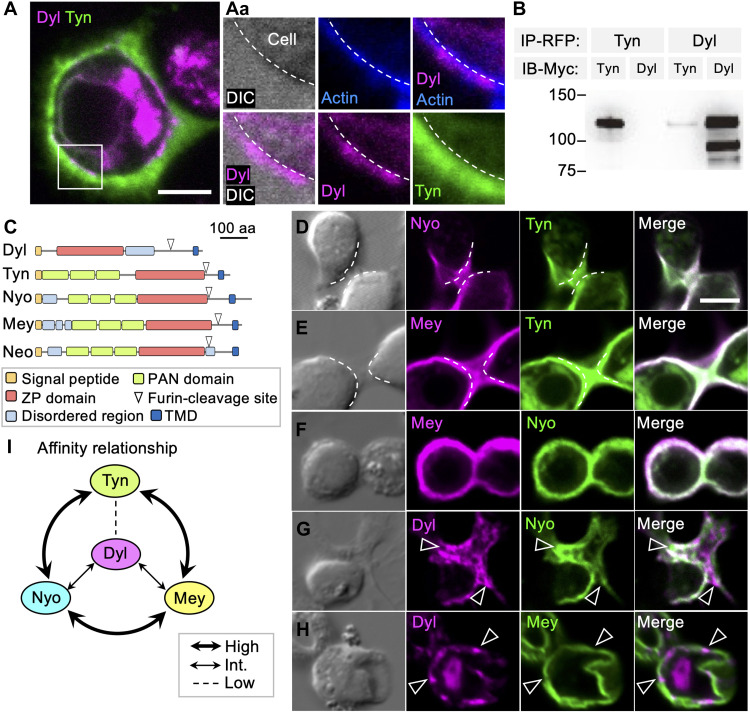
Self-organization of ZPD matrices in cultured cells. (**A**) A two-layered ECM structure on S2 cells overexpressing Myc::Tyn (green; stained with anti-Myc antibody) and mCherry::Dyl (magenta). (Aa) Enlarged views of the boxed region in (A). The dotted line indicates the cell membrane. (**B**) Immunoprecipitation assays. Tyn and Dyl constructs tagged with red fluorescent protein (RFP) or Myc were separately transfected to S2 cells, and the cells were cocultured in a pairwise manner. The culture supernatants were immunoprecipitated using anti-RFP antibody and were probed with anti-Myc antibody. Both Tyn and Dyl showed bands above 100 kDa, which represent the mature forms that have been cleaved at the Furin cleavage site shown in (C), resulting in the release of the extracellular parts of the proteins. The shorter form of Dyl appears to be a further degradation product. Homooligomers of Tyn and Dyl were abundantly detected. A weak band of RFP::Dyl and Myc::Tyn complex was also detected. No signal was detected from the reciprocal combination of RFP::Tyn and Myc::Dyl, due to higher expression level of the Tyn construct. (**C**) Structures of the five ZPD proteins. aa, amino acid; TMD, transmembrane domain. (**D** to **H**) Pairwise expression of mCherry::Tyn, mCherry::Dyl, mVenus::Nyo, and HA::Mey stained with anti–hemagglutinin (HA) antibody. Dotted lines indicate the cell boundaries. The open arrowheads in [(G) and (H)] show that the location of one of the proteins is underrepresented. This protein segregation was observed in 18/18, 7/11, and 11/11 cells expressing Dyl-Tyn, Dyl-Nyo, and Dyl-Mey pairs, respectively. No clear segregation of matrix proteins was observed in any of the Nyo-Tyn (0/10), Mey-Tyn (0/10), and Mey-Nyo (0/8) cells. (**I**) A diagram of the affinity relationship of ZPD proteins inferred from their mixing patterns. All protein tags were inserted immediately after the SP. Int., intermediate. Scale bar, 5 μm.

To uncover how ZPD proteins form ECM compartments on the olf hair cell, we expressed them in macrophage-derived *Drosophila* Schneider 2 (S2) cells. S2 cells express almost no ZPD transcript (*34*) and did not react to the antibody against Tyn. Separately expressed Tyn, Dyl, Nyo, and Mey were secreted and formed stable ECM structures. Those patterns were maintained when expressed in combinations (Fig. 2, A and D to H, and fig. S2). Neo was not secreted and was thus not analyzed further in S2 cells (fig. S2A). Tyn formed a loosely distributed matrix, and Dyl formed a thin shell-like layer covering the plasma membrane (Fig. 2A). Immunoprecipitation analysis of Tyn and Dyl, expressed in different S2 cells and collected from the culture supernatant, revealed that the two proteins predominantly formed homooligomers, with some forming heterooligomers (Fig. 2B).

Outside of the ZPD, Dyl has a disorganized region of flexible conformations, and Tyn has three plasminogen N-terminal (PAN) domains (Fig. 2C). Mutant proteins of Dyl without the disordered region (Dyl^ZP^) and Tyn without any PAN domains (Tyn^ZP^) formed ECM, and those lacking ZPDs (Dyl^ΔZP^ and Tyn^ΔZP^) failed to be integrated into ECM (fig. S2, B to E). Cleavage of the signal peptide (SP) and the furin-dependent C-terminal transmembrane region of Dyl^ZP^ and Tyn^ZP^ leaves ZPD only, suggesting that ZPD is necessary and sufficient for ECM formation. Coexpression of Tyn and Dyl^ZP^, and Tyn^ZP^ and Dyl formed distinct sorting patterns similar to those of the full-length Tyn-Dyl pair (Fig. 2A and fig. S2, F and G). These data indicate that Tyn and Dyl self-oligomerize and sort into distinct ECMs through the function of their ZPDs. The sorted patterns of Tyn and Dyl resembled the patterns of layer II and III cloud ECM and layer I, respectively, in the olf.

Pairwise expression of Tyn, Nyo, and Mey or expression of all three formed completely mixed ECMs (Fig. 2, D to F, and fig. S2A). On the other hand, coexpressed Nyo and Dyl and Mey and Dyl formed partially separated ECMs (Fig. 2, G and H). Tyn^ZP^ also mixed well with Nyo and Mey, and Dyl^ZP^ matrix sorted partially with Nyo and Mey (fig. S2, H to K). The results indicate that ZPD protein ECMs have distinct mutual affinities shown in Fig. 2I: The Tyn matrix has a high affinity for Nyo and Mey matrices, while its affinity for the Dyl matrix is low. Nyo and Mey matrices have an intermediate affinity for the Dyl matrix. Collectively, these results indicate that ZPDs of Tyn and Dyl are responsible for sorting and mixing patterns with other ZPD proteins. Together with differential expression and sequential secretion in different cells (fig. S1, G to K), cell-specific compartmentalized ECM patterns in the olf, mech, and sp are established.

Live imaging of S2 cells expressing the Tyn matrix showed the rapidly changing shape of the matrix and deformation of the shape of actively moving cells (Tyn; movie S1). Dyl matrix was also deformed and split upon cell division (Dyl; movie S2). A part of the cell cortex pinched by Tyn matrix accumulated filamentous actin (F-actin), suggesting high mechanical stress (fig. S2L). Tyn matrix covering the cell surface expanded and was ruptured during cell mass increase (movie S3 and fig. S2M). These data indicate that Tyn matrix and possibly Dyl matrix are elastic materials with a rigidity sufficient for deforming the interphase cell cortex [Young’s modulus ~ 1.0 kPa (*35*)].

### Cloud ECM is elastic

**Fig. 3.**
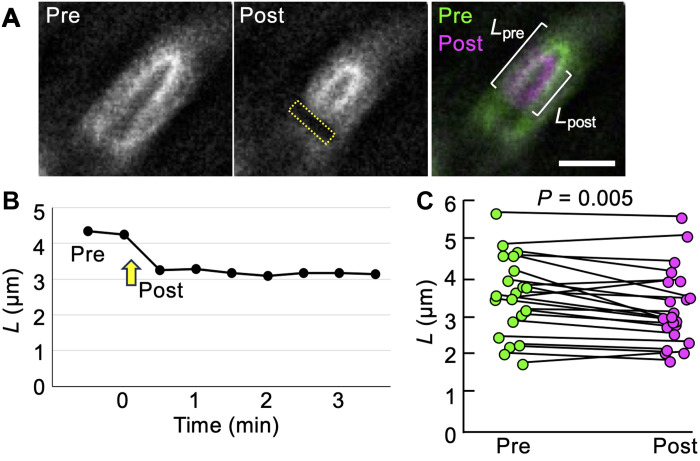
Laser ablation experiment to assess matrix elasticity. (**A**) mVenus::Tyn signal on the olf hair cells of prelaser (−0.5 min) and postlaser (0.5 min) illumination. The yellow dashed line indicates the area of laser illumination. The length (*L*) from the distal boundary of the ablation site to the tip of the strong mVenus::Tyn signal was measured both preablation (*L*_pre_) and postablation (*L*_post_). Scale bar, 5 μm. (**B**) A representative time course of *L*. The yellow arrow indicates the timing of ablation. A shortening of *L* was observed at 0.5 min postablation, with no further change thereafter. (**C**) Comparison of *L* before (−0.5 min) and after (0.5 min) ablation of olf cells showing the trend of *L* shortening (*n* = 22, four pupae). Statistical significance was determined using the Wilcoxon signed-rank test (*P* = 0.005).

To examine the physical properties of cloud ECM, we performed laser ablation experiments in olfactory sensilla. A two-photon laser was illuminated in a rectangular region across the base of the olf cell expressing mVenus::Tyn. Within 30 s after illumination, cloud ECM covering the distal side of the hair cell was rapidly contracted (Fig. 3, A and B). The contraction of cloud ECM was a general trend in multiple experiments (*n* = 22, four pupae; Fig. 3C). The data are consistent with the idea that cloud ECM is stretched by the pushing force applied by the olf hair cells. Laser-induced cell damage reduced the pushing force and resulted in the contraction of cloud ECM, indicating that cloud ECM is elastic.

### Dyl organizes envelope formation

**Fig. 4.**
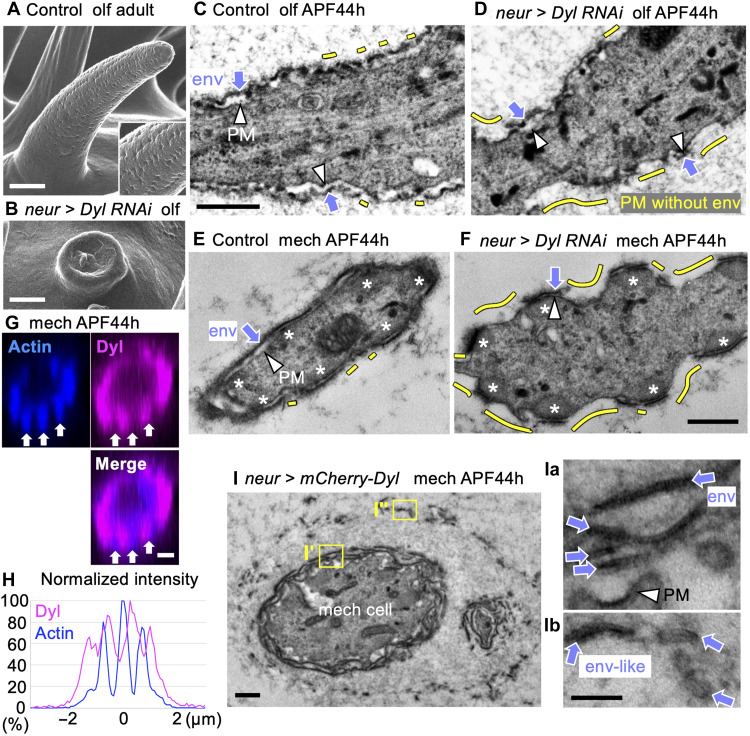
Dyl is required for env formation. (**A** and **B**) Olfactory bristles of normal (A) and Dyl–knocked-down (B) adult flies. The knockdown caused the collapse of bristle structure at the time of eclosion. (**C** and **D**) Longitudinal TEM views of olf at 44 hours APF (before the collapse). In the control (C), the PM (arrowheads) is covered by the env (arrows) except for small gaps (yellow lines; *n* = 2 flies). (D) Dyl RNAi caused a marked loss of env (yellow lines; Dyl RNAi-1, *n* = 2 flies). (**E** and **F**) Cross-sectional TEM views of mech in control (E) and Dyl–knocked-down flies (F). A marked loss of the env by Dyl suppression (yellow lines) occurred in the region free of actin bundles (asterisks). Dyl RNAi-2; *n* = 2 flies. (**G**) A fluorescent image of mScarlet::Dyl and stained actin in a cross section of mech. Arrows indicate the actin-positive sites. (**H**) The signal intensity profiles of Dyl and actin around the perimeter of mech hair cell in (G). (**I**) The multilayered env formation induced by Dyl overexpression (OE) in mech. *n* = 2 flies. (**Ia** and **Ib**) Enlargements of the boxed regions showing the parallel-lined structures (arrow) typical of the env (compare to Fig. 1C). Scale bars, 1 μm [(A), (B), and (G)], 100 nm [(Ia) and (Ib)], and 500 nm (others).

We next addressed the role of layer I component Dyl in cuticle formation. As shown in fig. S1B, Dyl was efficiently knocked down by *neur-gal4*–induced *dyl* RNAi. Resulting adults treated by two independent RNAi strains (dyl RNAi-1 and -2) showed the same, severe loss of the olfactory and mech in the adult maxillary palp (Fig. 4, A and B, and fig. S3, A and B). According to the previous report (*25*), knockdown of Dyl in mech on the thorax leads to short and translucent bristles due to postelongation collapse, rather than impaired outgrowth. In our maxillary palp model, hair elongation of both olf and mech proceeds normally at APF 44 hours, suggesting that the phenotype similarly results from postelongation instability. In this study, we found that the total amount of envelope on the olf shaft was reduced at 44 hours APF (Fig. 4, C and D) [envelope coverage: control: 96.8 and 97.7% *n* = 2; dyl RNAi-1 (*P{KK110997}VIE-260B*): 67.5 and 32.8%, *n* = 2; dyl RNAi-2 (*P{GD12695}v39022*): 68.2%, *n* = 1]. On the mech, the envelope was selectively lost in the region without the actin bundle (Fig. 4, E and F) (envelope coverage at actin-positive versus actin-negative regions: control: 100/100 and 98.4/99.7%, *n* = 2; dyl RNAi-1: 100/24.1%, *n* = 1; dyl RNAi-2: 88.5/25.9 and 76.6/32.2%, *n* = 2). Dyl accumulates as longitudinal stripes in the actin-negative region of the mech hair cells in APF 44 hours, as previously shown for the mech in the notum (Fig. 4, G and H) (*25*).

Overexpression of Dyl with *neur-gal4* caused truncation of the mech (fig. S3C). In the mech at 44 hours APF, overexpressed Dyl disrupted the endogenous layered pattern of Dyl, instead forming a thick ECM layer that covered the distal half of the cell (fig. S3, D, D′, and D″). At the corresponding site, multiple envelope-like layers surrounded the cell surface, extending up to 1 μm from the cell surface [Fig. 4, (I), (Ia), and (Ib)]. The results indicate that Dyl plays an essential role in the formation of an envelope around a subset of the plasma membrane. Its activity to induce multiple envelope-like structures indicates that Dyl has a key organizing role in envelope formation.

### Cloud ECM ensures regular nanopore formation

To understand the role of cloud ECM, we individually knocked down its components Tyn, Mey, Nyo, and Neo. As mentioned above, Tyn was lost around olf bristles expressing *tyn* RNAi, indicating that Tyn in cloud ECM is synthesized cell autonomously (fig. S1B). However, cloud ECM was still observed under transmission EM, and Mey, Nyo, and Neo were present around the olf hairs (fig. S4, A and B). Those olf hair cells at 44 hours APF increased width by 25% and length by 27% (1.98× volume increase) (fig. S4C; Tyn RNAi-1 *P{KK104282}VIE-260B*), suggesting that Tyn has a function to restrict the rapid growth of the olf around 44 hours APF (fig. S4D). However, the resultant adult olf cuticle still formed nanopores. Similarly, the appearance of Mey, Nyo, or Neo knockdown on the adult olf cuticle was indistinguishable from that of the control. The results indicate that cloud ECM is composed of multiple protein components, and the knockdown of individual ZPD proteins was insufficient to eliminate cloud ECM in toto.

**Fig. 5.**
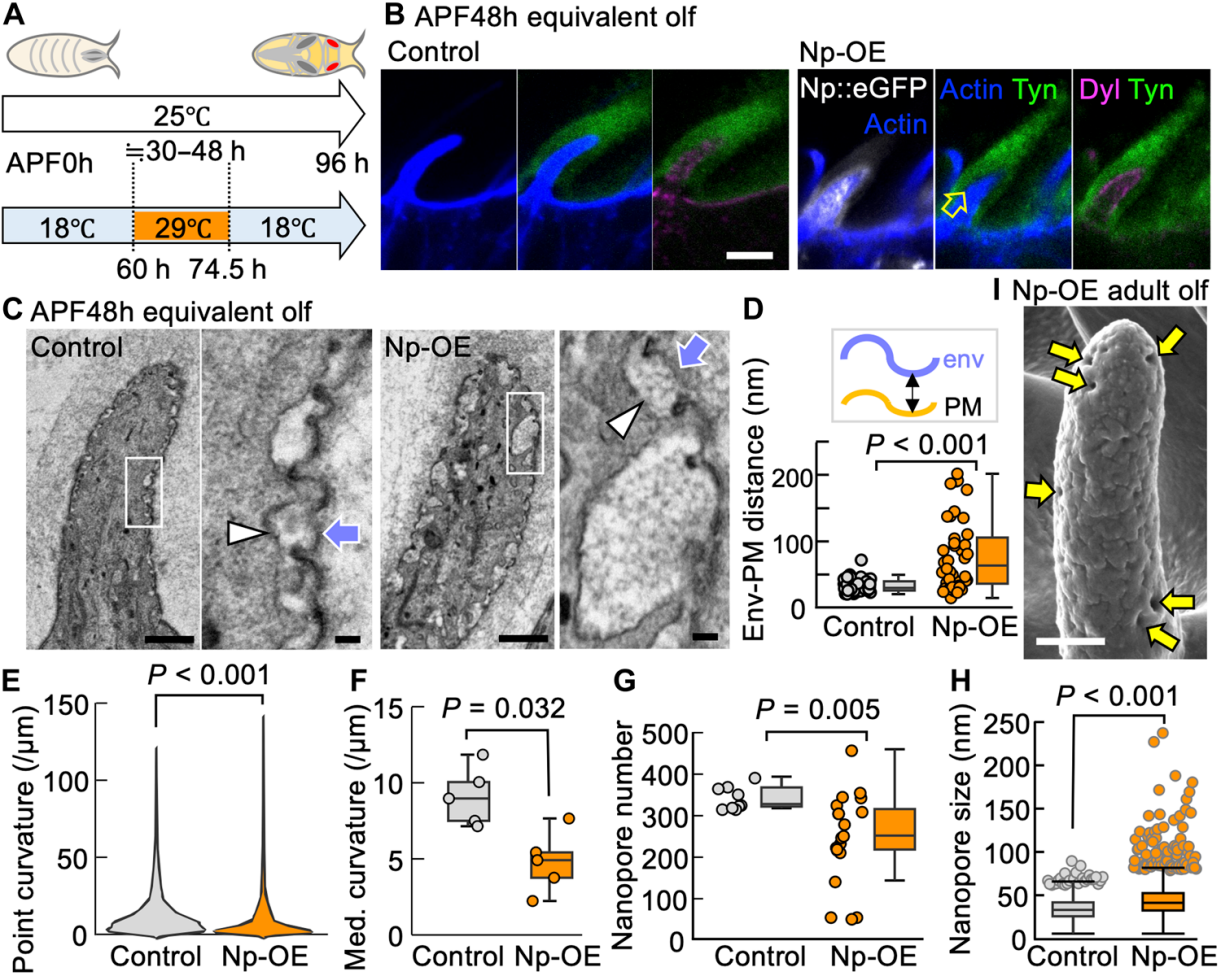
Np protease OE impaired cloud ECM and nanopore formation. (**A**) The temperature shift protocol of the Np-OE experiment. h, hours. (**B**) Olfactory bristles at 74.5 hours APF following the temperature shift procedure in (A), equivalent to 48 hours (h) APF at a constant incubation at 25°a, in the control [*neur-gal4, tub-gal80^ts^* without the upstream activation sequence (UAS) construct; left] and Np-overexpressing groups (*neur-gal4, tub-gal80^ts^ > UAS-Np::eGFP*; right). Np::eGFP (white), F-actin (blue), anti-Tyn (green), and anti-Dyl (magenta) signals are shown. The arrow indicates the gap between Tyn and the cell. Scale bar, 5 μm. (**C**) Longitudinal sections of olf by EM. The right panel (scale bar, 100 nm) is an enlarged view of the highlighted area in the left panel (scale bars, 1 μm) for each of the control and Np-OE. Arrowhead: PM. Arrow: env. (**D**) The distances between env and PM were measured at concave parts from TEM images (control: *n* = 42 and Np-OE: *n* = 41 concave parts from five hairs in a fly). (**E** and **F**) Point curvature of the env measured from TEM images using the Kappa plugin in Fiji (control: *n* = 39,552 points and Np-OE: *n* = 27,520 points) and the median curvature for each hair (*n* = 5 hairs from a fly for each group). Med., Median. (**G** and **H**) Quantification of nanopore number (control: *n* = 9 hairs from three flies and Np-OE: *n* = 20 hairs from seven flies) and size (control: *n* = 1550 pores from three flies and Np-OE: *n* = 2493 pores from seven flies; box plots with data points for the outliers were displayed) based on scanning EM (SEM) images. (**I**) SEM view of an olfactory bristle with Np-OE. Arrows indicate enlarged pores. Scale bar, 1 μm. Mann-Whitney *U* test was used for all statistical analyses.

As an alternative approach, we attempted to remove multiple ECM components by targeting protease expression. Among Stubble, Lumens interrupted, Notopleural (Np), and Tracheal-prostasin, which are known to degrade ZPD proteins Dumpy (Dpy) and Piopio (*36*, *37*), Np, a homolog of human matriptase, caused recognizable defects in the olf. Np::eGFP was expressed by *neur-gal4* and *tubulin-gal80^ts^* (a temperature-sensitive repressor of GAL4) during the time window corresponding to 30 to 48 hours APF at 25°C to avoid the lethality by its constitutive expression (Fig. 5A).

Np caused cleavage of Mey and Nyo, but not Tyn, in S2 cells (fig. S4E). Although the Tyn level was unchanged in the olf hairs as well, the distance of Tyn from the cell surface was expanded in Np-expressing olf (Fig. 5B and fig. S4F). In contrast, when Np was overexpressed in vivo, no obvious degradation of Nyo or Mey was observed by immunostaining. This discrepancy may be due to the differences in the relative amounts of Np and of Nyo or Mey compared to the in vitro setting. Dyl levels remained unchanged (Fig. 5B). We noted a ~55% increase in the olf width (2.25× volume increase; Fig. 5B and fig. S4G). EM views of Np-expressing olf cells showed that the distance between the envelope and plasma membrane was significantly increased (Fig. 5, C and D). The envelope took a partially wavy shape, but the curvature of the envelope decreased (Fig. 5, E and F). The Np expression did not compromise the outgrowth of the olf and mech (fig. S4H). However, the number of nanopores decreased, and the size became irregular, with large pores being observed (Fig. 5, G and H). Those results indicate that Np expression removes a subset of layer II ECM components, permitting overexpansion of the olf cell diameter and detachment of envelope from the cell membrane. Given the increased volume of olf hair cell observed upon Tyn knockdown (fig. S4C) and the contraction of cloud ECM following laser ablation (Fig. 3), we conclude that the cloud ECM provides the compressive force required for regular nanopore formation.

### Envelope-membrane proximity is essential for nanopore formation

**Fig. 6.**
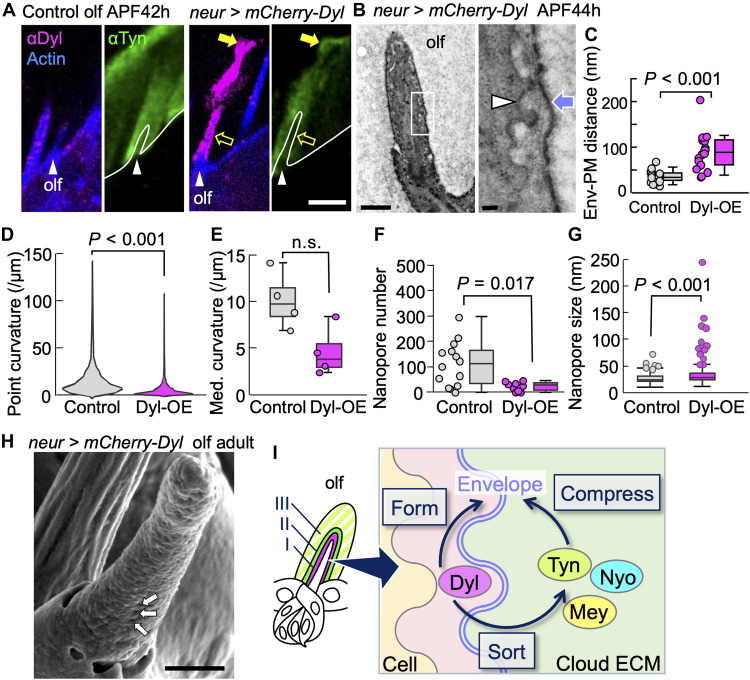
The env-PM proximity controls nanopore formation. (**A**) OE of Dyl in the olf hair cells. Anti-Dyl, anti-Tyn, and F-actin staining of control (left) and Dyl-OE (right) olf hair cells (arrowheads). Overexpressed Dyl covered the olf cell surface (open arrow) and accumulated at the tip (arrow). Scale bar, 5 μm. (**B**) An electron micrograph of Dyl-OE olf cell (left). Scale bar, 1 μm. The enlargement of the boxed area (right) shows a wavy PM (arrowhead) and a relatively straight env (arrow). Scale bar, 100 nm. (**C**) The distances between the env and PM (control: *n* = 19 and Dyl-OE: *n* = 16 from two hairs in two flies for each group). (**D** and **E**) Point curvature of the env (control: *n* = 14,080 points and Dyl-OE: *n* = 11,520 points) and the median curvature for each hair (*n* = 4 hairs from two flies for each group; *P* = 0.057) were calculated from TEM images. n.s., not significant. (**F** and **G**) Adult cuticle of Dyl-OE olf showed reduced nanopores (control: *n* = 14 hairs from five flies and Dyl-OE: *n* = 9 hairs from three flies), and irregular nanopore size (control: *n* = 790 pores from three flies and Dyl-OE: *n* = 108 pores from seven flies; box plots with data points for the outliers were displayed), as observed by SEM. (**H**) SEM view of a Dyl-OE olfactory bristle. Arrow: nanopore. Scale bar, 1 μm. Mann-Whitney *U* test was used for all statistical analyses. (**I**) A model of env formation and patterning by ZPD proteins. Layers I: Dyl; II: Tyn, Nyo, and Mey; and III: Tyn, Mey, and Neo are shown. Tyn, Nyo, and Mey are sorted to the outside of Dyl. The env layer forms at the interface between layers I (Dyl) and II + III (cloud ECM). Cloud ECM compresses the env layer to the PM. Juxtaposed envelope tracks the wavy shape of the PM.

In the olf at 42 to 44 hours APF, overexpressed Dyl formed tight coverage of the shaft cells (Fig. 6A, open arrow) and ectopic accumulation away from the olf hair cell (Fig. 6A, filled arrow). Endogenous Tyn was also present at the ectopic site, indicating that Dyl has the ability to deplete Tyn from its normal location. Overexpression of Dyl in the olf hair cells caused detachment of the envelope from the cell membrane (Fig. 6, B and C), as observed in Np overexpression (Fig. 5, C and D). Moreover, the envelope of Dyl-overexpressing olf often exhibited a flat appearance (Fig. 6, B, D, and E). Consistently, the number of nanopores decreased, and larger pores were observed (Fig. 6, F to H). The result indicates that the excess Dyl caused the displacement of the envelope from the plasma membrane and the loss of nanopore of the olf.

## DISCUSSION

In this work, we tested the hypothesis that the curved envelope of olf is formed by tracking the undulated plasma membrane (*10*). In support of this model, disruption of the close association between the envelope and plasma membrane by reducing the compressive force of layer II and III cloud ECM (Np overexpression) or thickening of layer I ECM (Dyl overexpression) caused loss or malformation of the nanopore. Apical ECM of insects undergoes rapid deposition and replacement during cuticle development (*38*–*40*). The ZPD proteins Dyl, Tyn, Nyo, and Mey form rapidly changing and cell type–specific aECM distributions during the early phase of sensory organ development, before the cuticle deposition. The functions of the ZPD aECM are twofold. Layer I occupies a thin territory of up to 125 nm above the plasma membrane: The envelope forms at the outer border of layer I. The self-organizing activity of Dyl to form a layered ECM structure in cultured cells may be responsible for layer I formation. Tyn is assembled outside of the surface of the Dyl ECM to form a two-layered organization. The prospective envelope components may be segregated from the Dyl layer and are assembled at the interface to construct the envelope in such a way that the amphipathic molecules align at the water-oil interface.

The second role of ZPD ECM is the formation of cloud ECM that occupies layers II and III of the olf. Proteolysis by Np caused the widening of the olf, suggesting that the ZPD ECM containing Tyn, Nyo, Mey, Neo, and possibly other proteins applies compressive force essential for nanopore formation. The high mixing activity of Tyn, Nyo, and Mey indicates that cloud ECM is a composite of multiple ZPD and other molecules. Removal of any one of those ZPD proteins did not cause a substantial change in the cuticle secretion and nanopore formation, indicating that the multiple molecular compositions of cloud ECM make the matrix robust against the fluctuation of ZPD protein quantity. The expression of transient ZPD matrices before the cuticle formation has been described as “precuticle” during vulval and sensory organ development in *caenorhabditis elegans* (*21*, *22*). Thus, the mechanical role of ZPD matrices in cuticle patterning reported here may play similar roles in a wide range of animal species.

The plasma membrane of the olf cells is highly undulated, whereas the plasma membrane in the mech and sp is flat (fig. S4, I, I″, and J). Two mechanisms can explain the difference in the membrane shape. First, cloud ECM covers the olf hair cell at the time of its outgrowth (38 to 39 hours APF) and is constantly stretched by the growing hair cell. Then cloud ECM applies a compressive force on the plasma membrane, which is increasing in the area size due to the extensive endoplasmic reticulum (ER) activity, and buckles to form a wavy pattern. The compressive cloud ECM may also restrict the envelope formation to the proximity of layer I ECM and plasma membrane so that the envelope formation follows the wavy undulation of the plasma membrane. The second mechanism is the presence of actin bundles underlying the plasma membrane of the mech and sp. In the mech, membranes attached to the actin bundles are flat, while those in the interbundle region are abundant with membrane protrusions (plasma membrane plaque; fig. S4I′) and often convoluted (*41*). Circumferential actin bundles in the tracheal tubule have been shown to suppress endocytosis (*42*). Those observations suggest the abundant actin filaments stabilize membrane dynamics and keep it flat. Low abundance of subcortical actin in the olf cells may thus permit frequent membrane deformation under abundant membrane supply, due to elevated ER activity in olf (*12*).

On the basis of the collective results, we propose a model whereby ZPD matrices form a wavy envelope layer required for nanopore formation (Fig. 6I). As described above, Dyl and Tyn form a two-layered organization, which, in turn, defines the interface where the envelope forms. Meanwhile, the cloud ECM, corresponding to layer II/III, appears to exert a compressive force on the olfactory hairs. This interpretation is supported by several key observations: (i) laser ablation of the olf hairs induces contraction of the cloud ECM, consistent with the release of pushing force from the cells; (ii) both Np overexpression and Tyn knockdown, which partially disrupt the cloud ECM, lead to increased hair cell volume; and (iii) olfactory hairs extend into the preformed cloud ECM and appear to be physically confined by its structure. Together, these findings support the idea that the cloud ECM provides mechanical compression to regulate hair cell shape. We propose that this compressive force shapes the envelope and layer I according to the undulated contours of the cell membrane, resulting in their characteristic wavy morphology.

ZPD proteins build various forms of matrix. Dpy forms tensile filaments that anchor a part of imaginal disc to direct tissue flow (*43*–*47*) and restrict tube elongation by forming luminal filaments in the trachea (*48*). Tyn, Nyo, and Mey form a three-dimensional cloud ECM, covering the cells to set up a compressive mechanical environment, and Dyl forms a sheet that organizes layer formation of the envelope. In cultured cells, ZPD of Dyl, Tyn, and Dpy are, in principle, sufficient for directing molecular assembly into the sheet and cloud (this study) and filamentous forms (*46*). Further investigations into ZPD structures may lead to an improved understanding of principles underlying modular matrix design based on the ZPD.

While the data presented here are consistent with the proposed model of cloud ECM in the cuticle nanopatterning, the complex composition of cloud ECM makes the genetic manipulation inefficient. Adult sensilla develop in the confined environment protected by the pupal cuticle that limits the direct physical measurement of ECM properties and super-resolution imaging. Nevertheless, the findings presented in this study will pave the way for uncovering the mechanisms of various other cuticle nanostructure formations and for the development of biomimetic technologies.

## MATERIALS AND METHODS

### Fly strains

*Drosophila melanogaster* strains were cultured with standard yeast-cornmeal-agar food at 25°C otherwise noted. For staging the pupae, we collected white pupae at APF 0 hours, allowed them to grow for a specific time, and then fixed them. w[*]; P{UAS-Np::eGFP} is a gift from M. Behr (*36*, *37*).

### Temperature control for Np overexpression experiment

Parental flies were maintained at 18°C, and offspring at the white pupal stage was collected. After 60 hours, the pupae were transferred to 29°C for 14.5 hours and then returned to 18°C. The heating time corresponds to around 30 to 48 hours APF period within the total 96-hour pupal stage at 25°C.

### Generation of expression vectors and transgenic flies

For the construction of expression vectors, the coding sequence of *dyl* or *tyn* with a tag right after the SP was introduced into pUASTattB plasmids. These plasmids were used as polymerase chain reaction templates for site-directed mutagenesis with the following primers, each paired with its reverse complement primer: “CAAGCACCTGCAGGTGGAGTACGGTCTGCCC” for pUASTattB-mCherry::Dyl^ΔZP^, “CCCCAGATTGGAGCCAAGGATGACCTGAGTGC” for pUASTattB-mCherry::Dyl^ZP^, “GCTATGATGTTTCTGTTGAATCTTTGGGGAGACGG” for pUASTattB-mCherry::Tyn^ΔZP^, and “GGAAGCGGAGGTTCCCCACTGATCGATCATGG” for pUASTattB-mCherry::Tyn^ZP^. pUASTattB-mVenus::Nyo and pUASTattB-HA::Mey were constructed and packaged by VectorBuilder (vectorbuilder.com). pUASTattB-mCherry::Dyl or pUASTattB-mCherry::Tyn (150 ng/μl each) was injected into the *y, v, nos-*φ*C31; attP40* strain, and the upstream activating sequence (UAS) strains were established.

### Generation of knock-in flies by genome editing

mVenus or mScarlet sandwiched with linkers was inserted after the sequence of SP in *dyl* or *tyn* gene with the use of the scarless gene editing system (https://flycrispr.org/scarless-gene-editing), a combination of CRISPR-Cas9–mediated homology-directed repair and precise excision by piggyBac (PBac) transposase as follows. The guide RNAs (gRNAs) for Dyl (GCCCATTTACGGTGCGCCCC) and Tyn (GTGATCGATCTTCGATGCAC) close to the insertion sites were designed with CRISPR Target Finder (*49*) (http://targetfinder.flycrispr.neuro.brown.edu) and cloned into pBFv-U6.2 vector (*50*) as described in our precious work (*29*). To generate donor plasmids, superfolder GFP (sfGFP) in pHD-sfGFP-ScarlessDsRed (Addgene, #80811) was first replaced by a fluorescent tag mVenus or mScarlet. A continuous genomic region, including the sequence from downstream of the SP to the gRNA site and its homology arms more than 500 base pairs for both sides, was amplified and inserted into a backbone vector. Last, the marker cassette flanked by transposon ends was inserted right after the SP in the modified backbone vector. Silent mutations were introduced into the gRNA sequence of the donor plasmid to avoid mutagenesis after the knock-in. The donor and gRNA constructs (150 ng/μl each) were injected into the *y[2] cho[2] v[1]; attP40{nos-Cas9}/CyO* strain. Once the tag was introduced into the genome, the DsRed marker was removed by crossing with w[1118]; CyO, P{Tub-PBac\T}2/wg[Sp-1]; l (3)*[*]/TM6B, Tb[1], and fly strains were established.

### Fluorescence microscopy

Pupae were removed from the pupal case and gently scratched to facilitate fixation and then immediately transferred to the fixation buffer [4% formaldehyde in phosphate-buffered saline, (PBS)]. After a few hours or one night at 4°C, pupal cuticles were removed, and collected head tissues were washed three times with 2% bovine serum albumin (BSA) and 0.2% of Triton X-100 in PBS, followed by antibody incubation. The head tissue underwent further dissection to isolate the maxillary palps with labellum and mounted with VECTASHIELD Mounting Medium (Vector Laboratories, H-1000 or H-2000). Images were taken using Olympus FV1000 with UPLSAPO60XW 60× numerical aperture–1.2 water-immersion lens. Rat anti-Dyl and rabbit anti-Tyn antibodies (1:300) were a gift from F. Payre (*20*). Peptides Cys-VATPNGSELPKPLS-OH for anti-Mey, Cys-VKQTNPRTNVTPSP-OH for anti-Nyo, and Cys-SLGSEEDSIYYDNA-OH for anti-Neo were used as immunogen and the ligands for affinity purification. Antibody production and purification were performed by Scrum Inc., Japan. Alexa Fluor Plus 405 Phalloidin (Thermo Fisher Scientific, catalog no. A30104) was used for actin staining.

### Laser ablation

We used mVenus::Tyn pupae at 44 to 48 hours APF for laser ablation experiments. The anterior part of pupal case was removed, and the maxillary palp was imaged from the ventral side using the confocal mode of Zeiss LSM 980 NLO inverted microscope. Time-lapse imaging was conducted at 30-s intervals for a duration of 5 to 10 min. Laser ablation of olf was performed after the second image acquisition using the two-photon laser. The length (*L*) from the distal boundary of the ablation site to the tip of the cellular protrusion was measured using Fiji. Measurements were taken at preablation (first frame, time: –0.5 min) and postablation (third frame, time: 0.5 min).

### Transmission EM

Samples were prepared following the previous work (*10*) with some modifications. Pupal head tissues were collected as described above and were fixed with the solution with 2.5% glutaraldehyde and 2% formaldehyde in 0.1 M cacodylate buffer (pH 7.4) overnight to up to 1 week at 4°C. The tissues were washed three times for 10 min in 0.1 M cacodylate buffer and postfixed in 2% OsO_4_, 1.5% (wt/vol) potassium ferrocyanide, 2 mM CaCl_2_, and 0.15 M sodium cacodylate buffer (pH 7.4) for 2 hours on ice with light shielding. The tissues were washed three times for 10 min in water, stained with 0.5% uranyl acetate at 4°C overnight, and washed twice in water for 15 min, followed by dehydration in a series of graded ethanol. The ethanol was replaced by propylene oxide and then resin (Epon 812, TAAB). The resin was polymerized at 60°C for more than 48 hours. After trimming, 60-nm-thickness ultrathin sections were cut with an ultramicrotome (RMC, MT-XL) and observed by JEM-1400Plus (JEOL). Two experiments (Fig. 1, D and H) were conducted by S.I. with modifications, and the data were from antennal large basiconic sensilla, which we consider are analogous to the olfactory bristles found in maxillary palp. First, for stronger staining of the envelope (Fig. 1D), the postfixation was followed by washes, 1% thiocarbohydrazide treatment for 1 hour at 60°C, additional washes, and the second postfixation for 1 hour on ice. Second, for RR staining (Fig. 1H), RR (0.5 mg/ml; Sigma-Aldrich, 00541; dissolved in water) was added to the postfixation solution.

### Immunoelectron microscopy

Pupae were immersed in a 4% PFA solution in PBS at 4°C for a few hours or overnight, and the isolated head tissues were then washed twice with PBS for 10 min each and treated with a solution of 1% BSA and 0.01% Triton X-100 in PBS for 10 min. Overnight incubation with a primary anti-GFP rabbit antibody (1:200; MBL, catalog no. 598) in the permeabilization solution was followed. The tissues were washed three times for 10 min and were incubated in a secondary goat anti-rabbit Fab’ Alexa Fluor 594 FluoroNanogold (1:200; Nanoprobes Inc., catalog no. 7304) for 2 hours at room temperature. After the wash with the permeabilization solution for 10 min and two 10-min washes in PBS, postfixation was performed using 0.5% glutaraldehyde in PBS for 30 min at 4°C. After washing the tissues twice in water for 10 min each, a gold enhancement treatment with GoldEnhance EM Plus (NAN, catalog no. 2114) was conducted for 5 min. Following another rinse in water, the tissues were dehydrated and embedded in resin and processed as described above. Ultrathin sections on grids were double stained using uranyl acetate and lead citrate, followed by washing and observation with JEM-1400Plus (JEOL).

### Scanning EM of the adult cuticles

Field emission scanning EM (FE-SEM) and helium ion microscopy (HIM) were performed on the basis of the previous report (*10*). Adult flies were collected in 70% ethanol and stored at room temperature. Head tissues were fixed and dehydrated as described for the transmission EM analysis but without uranyl acetate treatment. In the middle of this study, cacodylate buffer was replaced with PBS, as it yielded the same results. For drying, the samples were placed in a desiccator for about a week. Last, the samples were coated twice with osmium at 5 nm (Tennant 20, Meiwafosis) and observed by FE-SEM (JSM-IT700HR, JEOL) or HIM (ORION Plus, Carl Zeiss at the Nano-processing facility in AIST Tsukuba, Japan).

### Cell culture, transfection, staining, and observation

*Drosophila* S2 cells [RIKEN BioResource Center, RCB1153; RRID: CVCL_Z232 (*51*)] were cultured at 25°C in Schneider’s Drosophila medium (Thermo Fisher Scientific/Gibco, catalog no. 21720024) with 10% fetal bovine serum or in Sf-900 II SFM (Thermo Fisher Scientific/Gibco, catalog no. 10902096), both supplemented with Penicillin-Streptomycin (Gibco Pen Strep, #15140-122) at a dilution of 1:100. Transfection was performed using TransIT-Insect Transfection Reagent (Mirus Bio, catalog no. MIR6104), following the protocol provided but with a reduced amount of each DNA construct (50 to 200 ng; pWAGal4 and pUASTattB plasmids described above). Cultures were observed after 2 to 3 days. The staining of transfected cells was performed on coverslips submerged in the medium during culture. Alternatively, transfected cells were dropped onto coverslips and allowed to adhere for 30 to 60 min before the staining. The cells were washed with PBS and fixed with 4% paraformaldehyde (PFA) for 15 min at room temperature. The cells were washed again with PBS. For intracellular staining, cells were treated with a blocking solution (0.2% Triton X-100, 0.2% Tween 20, and 0.1% BSA in PBS) for 15 min. Primary and secondary antibodies were applied sequentially, each followed by three PBS washes. Antibodies used were rabbit anti-Myc polyclonal antibody (MBL, code 562), rat anti–hemagglutinin (HA) (Roche, 3F10), Alexa Fluor Plus 405 Phalloidin (Thermo Fisher Scientific, catalog no. A30104), goat anti-rabbit Immunoglobulin G (IgG) Alexa Fluor Plus 405 (Invitrogen), and goat anti-rat IgG STAR RED (Abberior, STRED-1007), all used at a dilution of 1:200. After staining, the cells were mounted and observed using an Olympus FV1000 microscope.

### Coimmunoprecipitation and Western blot analysis

S2 cells harboring a transgene pMT-mCherry::Tyn, pMT-mCherry::Dyl, pMT-MBP-Myc::Tyn, or pMT-MBP-Myc::Dyl were generated by cotransfection of pCopHygro, and each pMT plasmid was constructed using pMT/V5-HisA (Invirogen, V412020), followed by selection in Hygromycin B–containing medium (300 μg/ml; FUJIFILM, catalog no. 084-07681). A pair of transgenic cell lines at a density of 5 × 10^5^ cells/ml each was combined in 25-cm^2^ flasks, in a total of 5 ml of medium. Expression of the constructs was induced with 500 μM CuSO_4_ solution. Four days postinduction, when cells reached confluence, supernatant was collected and centrifuged at 1000 rpm. The supernatant was further centrifuged at 19,500*g*. The supernatant was mixed with 50 μl of anti–red fluorescent protein (RFP) or anti-Myc magnetic beads and incubated at 4°C for 1 hour with rotation. Beads were washed four times with 50 mM tris-HCl (pH 7.5) containing 150 mM NaCl and 0.05% NP-40. The proteins were eluted with 30 μl of Laemmli SDS sample buffer and heated at 95°C for 5 min before loading onto SDS–polyacrylamide gel electrophoresis (SDS-PAGE) gels. For Np cleavage assay, S2 cells were transfected with vectors UAS-mCherry::Tyn, UAS-mCherry::Dyl, UAS-HA::Mey, or UAS-mVenus::Nyo alone or with UAS-Np::eGFP (a gift from M. Behr). Supernatants (15 μl each) were analyzed by SDS-PAGE. Following SDS-PAGE, in both, proteins were transferred to polyvinylidene difluoride membranes using an iBlot 2 Dry Blotting System. Membranes were blocked and incubated with primary antibodies diluted in iBind solution, following the manufacturer’s recommendations. Signals were detected using Amersham ECL Prime Western Blotting Detection Reagent and visualized using the Davinch-Chemi chemiluminescence detection system. Antibodies used for the detection were anti-RFP-HRP-DirecT, anti-Myc-tag-HRP-DirecT, mouse monoclonal anti-HA-HRP-DirecT, rabbit anti-Nyo followed by anti-rabbit-IgG-HRP, and rabbit anti-GFP-HRP-DirecT (all from MBL, except for anti-Nyo generated in this study).

### Quantification and statistical analysis

ImageJ-Fiji (LOCI) processed and quantified images. JASP (JASP team) performed statistical analysis and plot drawing.

## References

[1] F. Orozco, B. Alfaro-González, Y. C. Ureña, K. Villalobos, A. Sanchez, F. Bravo, J. R. Vega, O. Argüello-Miranda, Nanobiodiversity: The potential of extracellular nanostructures. J. Renew. Mater. 5, 199–207 (2017).

[2] S. R. Shanbhag, B. Müller, R. A. Steinbrecht, Atlas of olfactory organs of *Drosophila melanogaster*: 1. Types, external organization, innervation and distribution of olfactory sensilla. Int. J. Insect Morphol. Embryol. 28, 377–397 (1999).

[3] M. Srinivasarao, Nano-optics in the biological world: Beetles, butterflies, birds, and moths. Chem. Rev. 99, 1935–1962 (1999). 11849015 10.1021/cr970080y

[4] G. Tan, J.-H. Lee, Y.-H. Lan, M.-K. Wei, L.-H. Peng, I.-C. Cheng, S.-T. Wu, Broadband antireflection film with moth-eye-like structure for flexible display applications. Optica 4, 678–683 (2017).

[5] M. A. Samaha, H. V. Tafreshi, M. Gad-el-Hak, Superhydrophobic surfaces: From the lotus leaf to the submarine. Comptes Rendus Mecanique 340, 18–34 (2012).

[6] S. S. Latthe, C. Terashima, K. Nakata, A. Fujishima, Superhydrophobic surfaces developed by mimicking hierarchical surface morphology of lotus leaf. Molecules 19, 4256–4283 (2014). 24714190 10.3390/molecules19044256 PMC6270765

[7] B. Bhushan, Biomimetics: Lessons from nature–An overview. Philos. Trans. A Math. Phys. Eng. Sci. 367, 1445–1486 (2009). 19324719 10.1098/rsta.2009.0011

[8] V. B. Wigglesworth, The insect cuticle. Biol. Rev. 23, 408–451 (1948). 18122255 10.1111/j.1469-185x.1948.tb00566.x

[9] M. Locke, Pore canals and related structures in insect cuticle. J. Biophys. Biochem. Cytol. 10, 589–618 (1961). 13762980 10.1083/jcb.10.4.589 PMC2225106

[10] T. Ando, S. Sekine, S. Inagaki, K. Misaki, L. Badel, H. Moriya, M. M. Sami, Y. Itakura, T. Chihara, H. Kazama, S. Yonemura, S. Hayashi, Nanopore formation in the cuticle of an insect olfactory sensillum. Curr. Biol. 29, 1512–1520.e6 (2019). 31006566 10.1016/j.cub.2019.03.043

[11] Z. Sun, S. Inagaki, K. Miyoshi, K. Saito, S. Hayashi, Osiris gene family defines the cuticle nanopatterns of *Drosophila*. Genetics 227, iyae065 (2024). 38652268 10.1093/genetics/iyae065 PMC11151929

[12] S. Inagaki, H. Wada, T. Itabashi, Y. Itakura, R. Nakagawa, L. Chen, K. Murata, A. H. Iwane, S. Hayashi, Endoplasmic reticulum patterns insect cuticle nanostructure. *J. Cell Biol.* 10.1083/jcb.202503127 (2025). 10.1083/jcb.202503127 PMC12755865 41474612

[13] L. Jovine, C. C. Darie, E. S. Litscher, P. M. Wassarman, Zona pellucida domain proteins. Annu. Rev. Biochem. 74, 83–114 (2005). 15952882 10.1146/annurev.biochem.74.082803.133039

[14] S. Plaza, H. Chanut-Delalande, I. Fernandes, P. M. Wassarman, F. Payre, From A to Z: Apical structures and zona pellucida-domain proteins. Trends Cell Biol. 20, 524–532 (2010). 20598543 10.1016/j.tcb.2010.06.002

[15] P. Bork, C. Sander, A large domain common to sperm receptors (Zp2 and Zp3) and TGF-β type III receptor. FEBS Lett. 300, 237–240 (1992). 1313375 10.1016/0014-5793(92)80853-9

[16] L. Jovine, H. Qi, Z. Williams, E. Litscher, P. M. Wassarman, The ZP domain is a conserved module for polymerization of extracellular proteins. Nat. Cell Biol. 4, 457–461 (2002). 12021773 10.1038/ncb802

[17] L. F. Sobala, P. N. Adler, The gene expression program for the formation of wing cuticle in *Drosophila*. PLOS Genet. 12, e1006100 (2016). 27232182 10.1371/journal.pgen.1006100 PMC4883753

[18] A. Jaźwińska, M. Affolter, A family of genes encoding zona pellucida (ZP) domain proteins is expressed in various epithelial tissues during *Drosophila* embryogenesis. Gene Expr. Patterns 4, 413–421 (2004). 15183308 10.1016/j.modgep.2004.01.003

[19] A. Jaźwińska, C. Ribeiro, M. Affolter, Epithelial tube morphogenesis during *Drosophila* tracheal development requires Piopio, a luminal ZP protein. Nat. Cell Biol. 5, 895–901 (2003). 12973360 10.1038/ncb1049

[20] I. Fernandes, H. Chanut-Delalande, P. Ferrer, Y. Latapie, L. Waltzer, M. Affolter, F. Payre, S. Plaza, Zona pellucida domain proteins remodel the apical compartment for localized cell shape changes. Dev. Cell 18, 64–76 (2010). 20152178 10.1016/j.devcel.2009.11.009

[21] J. D. Cohen, A. P. Sparacio, A. C. Belfi, R. Forman-Rubinsky, D. H. Hall, H. M. Maul-Newby, A. R. Frand, M. V. Sundaram, A multi-layered and dynamic apical extracellular matrix shapes the vulva lumen in *Caenorhabditis elegans*. eLife 9, e57874 (2020). 32975517 10.7554/eLife.57874 PMC7544507

[22] H. K. Gill, J. D. Cohen, J. Ayala-Figueroa, R. Forman-Rubinsky, C. Poggioli, K. Bickard, J. M. Parry, P. Pu, D. H. Hall, M. V. Sundaram, Integrity of narrow epithelial tubes in the *C. elegans* excretory system requires a transient luminal matrix. PLOS Genet. 12, e1006205 (2016). 27482894 10.1371/journal.pgen.1006205 PMC4970718

[23] M. V. Sundaram, N. Pujol, The *Caenorhabditis elegans* cuticle and precuticle: A model for studying dynamic apical extracellular matrices in vivo. Genetics 227, iyae072 (2024). 38995735 10.1093/genetics/iyae072 PMC11304992

[24] W. Fung, I. Kolotuev, M. G. Heiman, Specialized structure and function of the apical extracellular matrix at sense organs. Cells Dev. 179, 203942 (2024). 39067521 10.1016/j.cdev.2024.203942 PMC11346620

[25] R. Nagaraj, P. N. Adler, Dusky-like functions as a Rab11 effector for the deposition of cuticle during *Drosophila* bristle development. Development 139, 906–916 (2012). 22278919 10.1242/dev.074252 PMC3274354

[26] P. N. Adler, L. F. Sobala, D. S. Thom, R. Nagaraj, *dusky-like* is required to maintain the integrity and planar cell polarity of hairs during the development of the *Drosophila* wing. Dev. Biol. 379, 76–91 (2013). 23623898 10.1016/j.ydbio.2013.04.012 PMC3686509

[27] N. Ghosh, J. E. Treisman, Apical cell expansion maintained by Dusky-like establishes a scaffold for corneal lens morphogenesis. Sci. Adv. 10, eado4167 (2024). 39167639 10.1126/sciadv.ado4167 PMC11338227

[28] Y. Wang, J. Berger, B. Moussian, Trynity models a tube valve in the *Drosophila* larval airway system. Dev. Biol. 437, 75–83 (2018). 29518377 10.1016/j.ydbio.2018.02.019

[29] Y. Itakura, S. Inagaki, H. Wada, S. Hayashi, Trynity controls epidermal barrier function and respiratory tube maturation in *Drosophila* by modulating apical extracellular matrix nano-patterning. PLOS ONE 13, e0209058 (2018). 30576352 10.1371/journal.pone.0209058 PMC6303098

[30] W. Fung, T. M. Tan, I. Kolotuev, M. G. Heiman, A sex-specific switch in a single glial cell patterns the apical extracellular matrix. Curr. Biol. 33, 4174–4186.e7 (2023). 37708887 10.1016/j.cub.2023.08.046 PMC10578079

[31] J. H. Luft, Electron microscopy of cell extraneous coats as revealed by ruthenium red staining. J. Cell Biol. 23, 54–55 (1964).

[32] Y. Bellaïche, M. Gho, J. A. Kaltschmidt, A. H. Brand, F. Schweisguth, Frizzled regulates localization of cell-fate determinants and mitotic spindle rotation during asymmetric cell division. Nat. Cell Biol. 3, 50–57 (2001). 11146626 10.1038/35050558

[33] T. Otani, K. Oshima, S. Onishi, M. Takeda, K. Shinmyozu, S. Yonemura, S. Hayashi, IKKɛ regulates cell elongation through recycling endosome shuttling. Dev. Cell 20, 219–232 (2011). 21316589 10.1016/j.devcel.2011.02.001

[34] modENCODE Consortium, S. Roy, J. Ernst, P. V. Kharchenko, P. Kheradpour, N. Negre, M. L. Eaton, J. M. Landolin, C. A. Bristow, L. Ma, M. F. Lin, S. Washietl, B. I. Arshinoff, F. Ay, P. E. Meyer, N. Robine, N. L. Washington, L. D. Stefano, E. Berezikov, C. D. Brown, R. Candeias, J. W. Carlson, A. Carr, I. Jungreis, D. Marbach, R. Sealfon, M. Y. Tolstorukov, S. Will, A. A. Alekseyenko, C. Artieri, B. W. Booth, A. N. Brooks, Q. Dai, C. A. Davis, M. O. Duff, X. Feng, A. A. Gorchakov, T. Gu, J. G. Henikoff, P. Kapranov, R. Li, H. K. MacAlpine, J. Malone, A. Minoda, J. Nordman, K. Okamura, M. Perry, S. K. Powell, N. C. Riddle, A. Sakai, A. Samsonova, J. E. Sandler, Y. B. Schwartz, N. Sher, R. Spokony, D. Sturgill, M. van Baren, K. H. Wan, L. Yang, C. Yu, E. Feingold, P. Good, M. Guyer, R. Lowdon, K. Ahmad, J. Andrews, B. Berger, S. E. Brenner, M. R. Brent, L. Cherbas, S. C. R. Elgin, T. R. Gingeras, R. Grossman, R. A. Hoskins, T. C. Kaufman, W. Kent, M. I. Kuroda, T. Orr-Weaver, N. Perrimon, V. Pirrotta, J. W. Posakony, B. Ren, S. Russell, P. Cherbas, B. R. Graveley, S. Lewis, G. Micklem, B. Oliver, P. J. Park, S. E. Celniker, S. Henikoff, G. H. Karpen, E. C. Lai, D. M. MacAlpine, L. D. Stein, K. P. White, M. Kellis, Identification of functional elements and regulatory circuits by *Drosophila* modENCODE. Science 330, 1787–1797 (2010). 21177974 10.1126/science.1198374 PMC3192495

[35] P. Kunda, A. E. Pelling, T. Liu, B. Baum, Moesin controls cortical rigidity, cell rounding, and spindle morphogenesis during mitosis. Curr. Biol. 18, 91–101 (2008). 18207738 10.1016/j.cub.2007.12.051

[36] L. Drees, T. Königsmann, M. H. J. Jaspers, R. Pflanz, D. Riedel, R. Schuh, Conserved function of the matriptase-prostasin proteolytic cascade during epithelial morphogenesis. PLOS Genet. 15, e1007882 (2019). 30601807 10.1371/journal.pgen.1007882 PMC6331135

[37] L. Drees, S. Schneider, D. Riedel, R. Schuh, M. Behr, The proteolysis of ZP proteins is essential to control cell membrane structure and integrity of developing tracheal tubes in *Drosophila*. eLife 12, e91079 (2023). 37872795 10.7554/eLife.91079 PMC10597583

[38] A. Tonning, J. Hemphälä, E. Tång, U. Nannmark, C. Samakovlis, A. Uv, A transient luminal chitinous matrix is required to model epithelial tube diameter in the *Drosophila* trachea. Dev. Cell 9, 423–430 (2005). 16139230 10.1016/j.devcel.2005.07.012

[39] V. Tsarouhas, K. A. Senti, S. A. Jayaram, K. Tiklová, J. Hemphälä, J. Adler, C. Samakovlis, Sequential pulses of apical epithelial secretion and endocytosis drive airway maturation in *Drosophila*. Dev. Cell 13, 214–225 (2007). 17681133 10.1016/j.devcel.2007.06.008

[40] B. Moussian, E. Tång, A. Tonning, S. Helms, H. Schwarz, C. Nüsslein-Volhard, A. E. Uv, *Drosophila* knickkopf and retroactive are needed for epithelial tube growth and cuticle differentiation through their specific requirement for chitin filament organization. Development 133, 163–171 (2006). 16339194 10.1242/dev.02177

[41] L. G. Tilney, P. Connelly, S. Smith, G. M. Guild, F-actin bundles in *Drosophila* bristles are assembled from modules composed of short filaments. J. Cell Biol. 135, 1291–1308 (1996). 8947552 10.1083/jcb.135.5.1291 PMC2121084

[42] V. Tsarouhas, D. Liu, G. Tsikala, A. Fedoseienko, K. Zinn, R. Matsuda, D. D. Billadeau, C. Samakovlis, WASH phosphorylation balances endosomal versus cortical actin network integrities during epithelial morphogenesis. Nat. Commun. 10, 2193 (2019). 31097705 10.1038/s41467-019-10229-6 PMC6522504

[43] R. Etournay, M. Popović, M. Merkel, A. Nandi, C. Blasse, B. Aigouy, H. Brandl, G. Myers, G. Salbreux, F. Jülicher, S. Eaton, Interplay of cell dynamics and epithelial tension during morphogenesis of the *Drosophila* pupal wing. eLife 4, e07090 (2015). 26102528 10.7554/eLife.07090 PMC4574473

[44] A. Tsuboi, K. Fujimoto, T. Kondo, Spatiotemporal remodeling of extracellular matrix orients epithelial sheet folding. Sci. Adv. 9, eadh2154 (2023). 37656799 10.1126/sciadv.adh2154 PMC10854429

[45] T. Ayukawa, M. Akiyama, Y. Hozumi, K. Ishimoto, J. Sasaki, H. Senoo, T. Sasaki, M. Yamazaki, Tissue flow regulates planar cell polarity independently of the Frizzled core pathway. Cell Rep. 40, 111388 (2022). 36130497 10.1016/j.celrep.2022.111388

[46] W.-C. Chu, S. Hayashi, Mechano-chemical enforcement of tendon apical ECM into nano-filaments during *Drosophila* flight muscle development. Curr. Biol. 31, 1366–1378.e7 (2021). 33545042 10.1016/j.cub.2021.01.010

[47] R. P. Ray, A. Matamoro-Vidal, P. S. Ribeiro, N. Tapon, D. Houle, I. Salazar-Ciudad, B. J. Thompson, Patterned anchorage to the apical extracellular matrix defines tissue shape in the developing appendages of *Drosophila*. Dev. Cell 34, 310–322 (2015). 26190146 10.1016/j.devcel.2015.06.019 PMC4539345

[48] B. Dong, E. Hannezo, S. Hayashi, Balance between apical membrane growth and luminal matrix resistance determines epithelial tubule shape. Cell Rep. 7, 941–950 (2014). 24794438 10.1016/j.celrep.2014.03.066

[49] S. J. Gratz, F. P. Ukken, C. D. Rubinstein, G. Thiede, L. K. Donohue, A. M. Cummings, K. M. Oconnor-Giles, Highly specific and efficient CRISPR/Cas9-catalyzed homology-directed repair in *Drosophila*. Genetics 196, 961–971 (2014). 24478335 10.1534/genetics.113.160713 PMC3982687

[50] S. Kondo, R. Ueda, Highly improved gene targeting by germline-specific Cas9 expression in *Drosophila*. Genetics 195, 715–721 (2013). 24002648 10.1534/genetics.113.156737 PMC3813859

[51] I. Schneider, Cell lines derived from late embryonic stages of *Drosophila melanogaster*. Development 27, 353–365 (1972). 4625067

