# Supplementary material for: Mechanical control of the insect extracellular matrix nanostructure
Source: Sci Adv. 2026 Jan 2;12(1):eadw5022. doi: 10.1126/sciadv.adw5022 (PMC12758534; doi:10.1126/sciadv.adw5022)
Supplement: Supplementary file 1 — Figs. S1 to S4 Legends for movies S1 to S3 Legend for data S1 [file sciadv.adw5022_sm.pdf]

Science Advances
AAAS

Supplementary Materials for

Mechanical control of the insect extracellular matrix nanostructure

Yuki Itakura *et al.*

Corresponding author: Shigeo Hayashi, shigeo.hayashi@riken.jp

Sci. Adv. **12**, eadw5022 (2026)
DOI: 10.1126/sciadv.adw5022

The PDF file includes:

Figs. S1 to S4
Legends for movies S1 to S3
Legend for data S1

Other Supplementary Material for this manuscript includes the following:

Movies S1 to S3
Data S1

Fig. S1. Cell-specific apical ECM structures. (A) Distribution of anti-Dyl and anti-Tyn signals in the maxillary palp 44 h APF. Dyl is prominent on the spinule (sp, open arrow) and the mechanosensory hair cells (mech, arrow). Lower level of Dyl was also detected on the olfactory hair cell (olf, arrowhead) and epidermis. Tyn is abundant around the olf, the socket part of mech, and the exuvial space. **(B)** Specificity of Dyl and Tyn RNAi. Fluorescence images of mScarlet::Dyl and mVenus::Tyn knock in alleles at 44 h APF under three conditions: control, Dyl RNAi, and Tyn RNAi. RNAi was targeted to the olf, but not to the sp or epidermis. **(C)** Anti-Nyo, anti-Mey, and anti-Neo signals on olf, sp, and mech. The asterisk indicates the socket of the mech. **(D** and **E)** Anti-Nyo or anti-Neo staining together with endogenous mVenus::Tyn signals is shown. At 44 h APF, Nyo and Neo are localized to the proximal and distal regions of the Tyn ECM, respectively. **(F)** Anti-Mey signal is primarily located proximally, but a weak signal is also observed in the distal region. **(G)** A diagram of the layered ECM structures on olf, mech, spinule hair cells and epidermis at 38 and 44 h APF. The dotted areas indicate weak expression. **(H** to **J)** At 38 h APF, mVenus::Tyn, anti-Mey, anti-Neo, and anti-Dyl signals are present on olf (yellow arrowheads), while anti-Nyo signal is absent. Spinules have not grown yet in this stage. **(K)** At 39 h APF, anti-Nyo signal is observed at the proximal region (layer I) of olf. Spinules first appear at this stage with anti-Dyl signals on their tip (open arrows). Scale bar: 5 μm.

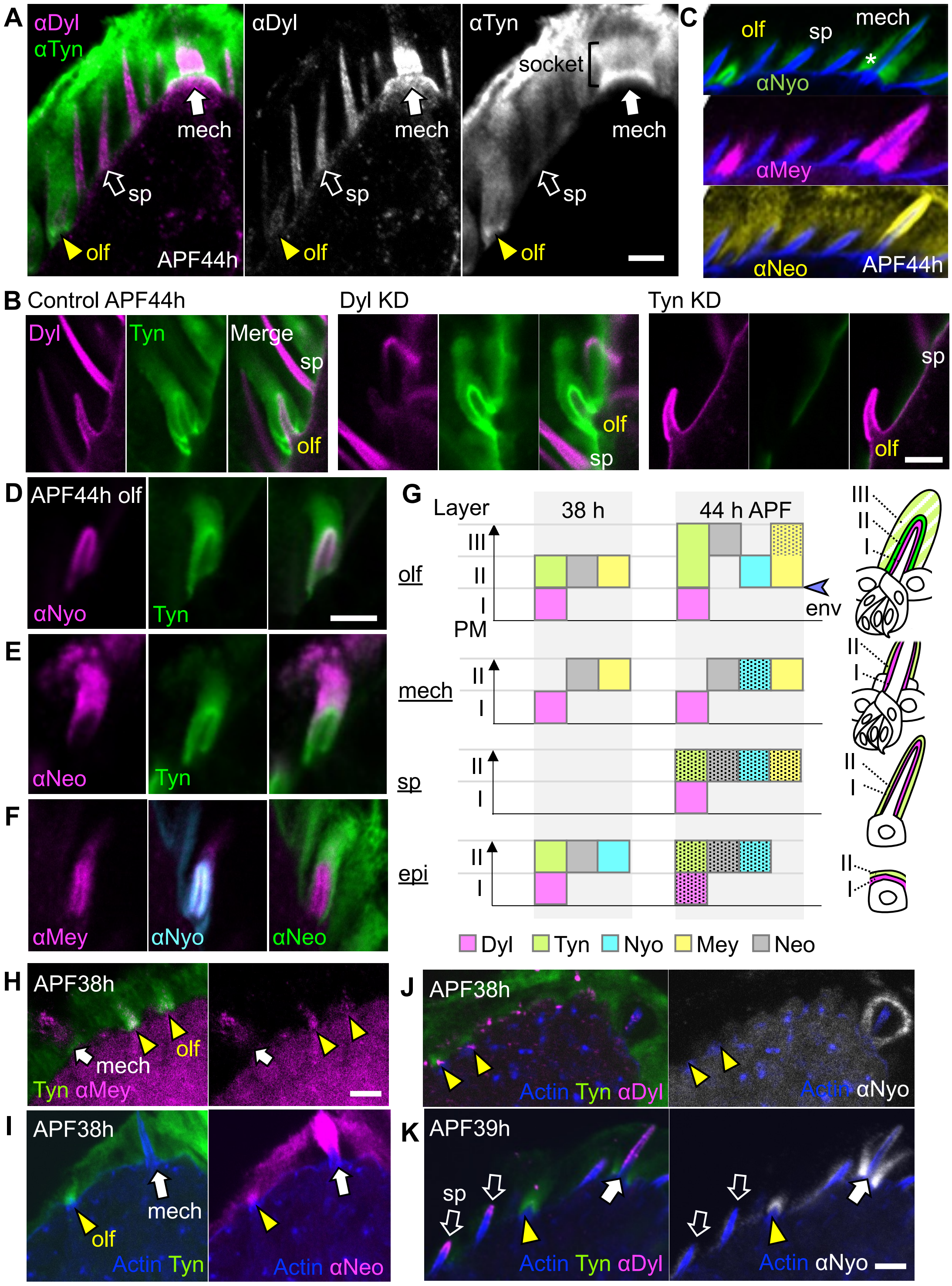
A
αDyl
αTyn
mech
sp
olf
APF44h
αDyl
αTyn
socket
C
olf
sp
mech
αNyo
αMey
αNeo
APF44h
B Control APF44h
Dyl
Tyn
Merge
Dyl KD
Tyn KD
D APF44h olf
αNyo
Tyn
E
αNeo
F
αMey
αNyo
αNeo
G
Layer
38 h
44 h APF
III
II
I
PM
olf
mech
sp
epi
env
Dyl
Tyn
Nyo
Mey
Neo
H APF38h
Tyn αMey
I APF38h
Actin Tyn
Actin αNeo
J APF38h
Actin Tyn αDyl
Actin αNyo
K APF39h
Actin Tyn αDyl
Actin αNyo

Fig. S2. Elastic and mixing properties of ZPD matrices. (**A** to **K**), Expression and mixing property of various forms of ZPD proteins. **(A)** Co-expressed mCherry::Tyn, mVenus::Nyo, and HA::Mey formed well-mixed ECM structure. Myc::Neo was not integrated into the ECM. **(B** and **C)** Myc::Dyl and mCherry::Dyl^{ZP} (Dyl without disordered region), and Myc::Tyn and mCherry::Tyn^{ZP} (Tyn without PAN domains) formed well-mixed ECM. **(D** and **E)** mCherry::Dyl$^{\Delta ZP}$ and mCherry::Tyn$^{\Delta ZP}$ lacking ZPDs did not form ECM, even in the presence of their full-length forms. **(F** and **G)** Combinations of Myc::Tyn and mCherry::Dyl^{ZP}, and mCherry::Tyn^{ZP} and Myc::Dyl, formed segregated ECM as the combination of the full-length forms of each protein. **(H** and **I)** mCherry::Tyn^{ZP} co-expressed with mVenus::Nyo or HA::Mey. **(J** and **K)** mCherry::Dyl^{ZP} co-expressed with mVenus::Nyo or HA::Mey. Tyn^{ZP} and Dyl^{ZP} exhibited the same co-localization and separation behaviors with Nyo and Mey, as their full-length forms. **(L)** Tyn matrix deforms cell membrane (arrow). Note the high accumulation of F-actin in the protruded cell membrane in the Tyn matrix (arrow). **(M)** Myc::Tyn matrix surrounding S2 cell was imaged for over 7 hours. It was expanded by the growing S2 cell (0 min: start of expansion). Image of 70 min shows expanded Tyn matrix (compare to the yellow dotted line showing the cell perimeter at 0 min, the starting point of Movie S3). The Tyn matrix punctuated at 90 min (yellow arrowheads), and shrunk (125 min, as compared to the yellow dotted line of cell perimeter at 70 min). The cell membrane protruded from the broken ECM as indicated by the white arrowhead in the DIC (Differential Interference Contrast) channel at 125 min. The length of Tyn ECM perimeter was shown in the rightmost panel. Scale bars: 5 μm.

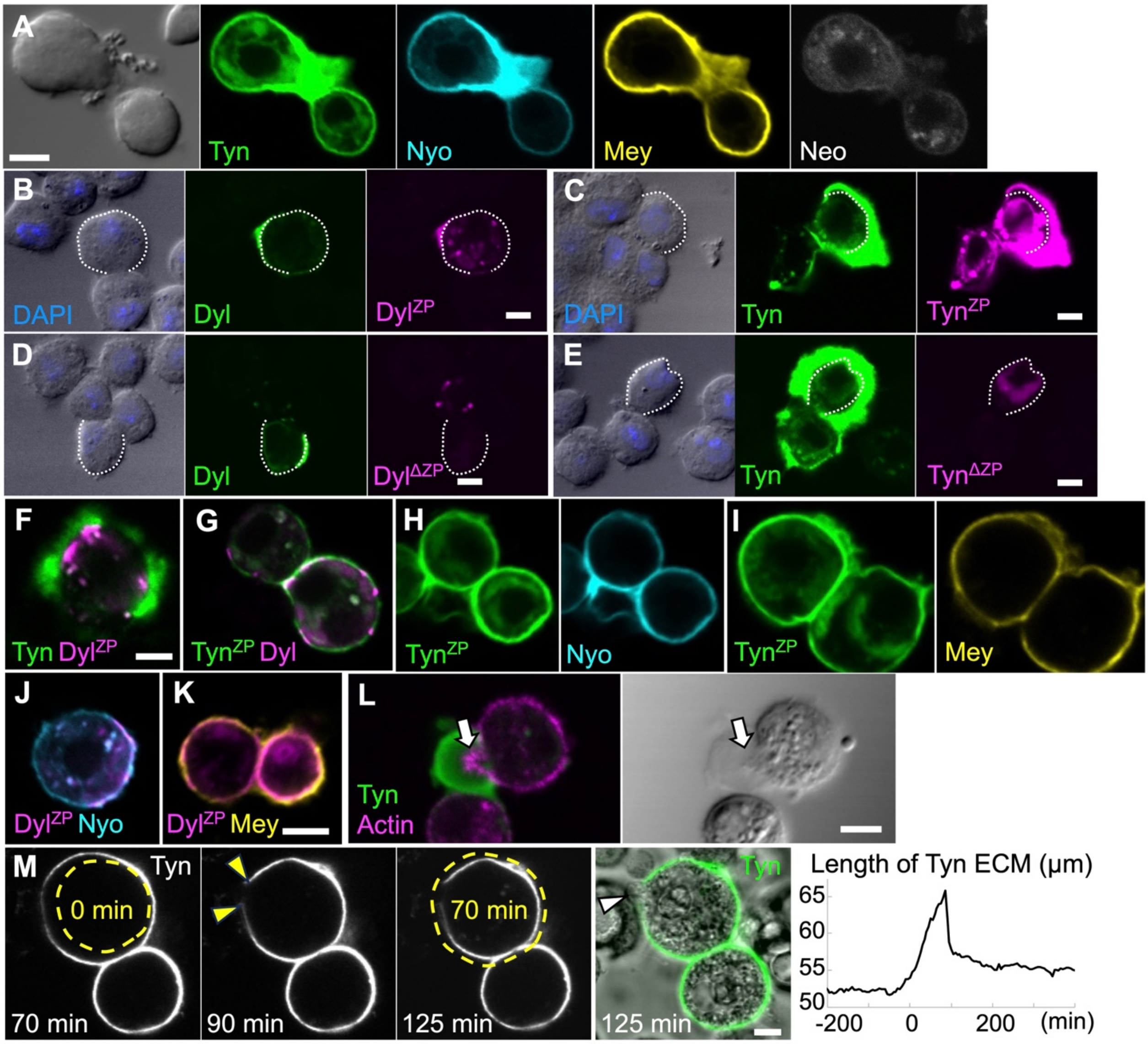

A
Tyn
Nyo
Mey
Neo
B
DAPI
Dyl
Dyl^ZP
C
DAPI
Tyn
Tyn^ZP
D
Dyl
Dyl^ΔZP
E
Tyn
Tyn^ΔZP
F
Tyn Dyl^ZP
G
Tyn^ZP Dyl
H
Tyn^ZP
Nyo
I
Tyn^ZP
Mey
J
Dyl^ZP Nyo
K
Dyl^ZP Mey
L
Tyn
Actin
M
Tyn
0 min
70 min
70 min
90 min
125 min
Tyn
125 min
Length of Tyn ECM (μm)
65
60
55
50
-200
0
200
(min)

Fig. S3. Roles of Dyl on bristle structures. (A to **C)** Olfactory (arrowheads) and mechanosensory (arrows) bristles on the adult maxillary palp. **(A)** Control fly. **(B)** Dyl RNAi driven by *neur-gal4* caused the shortening of mech (yellow arrows) and collapse of olf (yellow arrowheads, an enlarged view is shown in Fig. 4B). **(C)** With mCherry::Dyl overexpression, many of the mechanosensory bristles showed shortening or disorganized surface structure (yellow arrows). The surface structure of the olf bristles (arrowheads) was disorganized, as shown in the enlarged view in Fig. 6H. **(D)** Overexpressed mCherry::Dyl (magenta) accumulated around the distal part of the mechanosensory bristle (distal to the open arrow). The stripe pattern of actin bundles (white signal) is intact. mCherry::Dyl and immunolabeling of Dyl (green) that labels both overexpressed and endogenous Dyl exhibited similar disorganized patterns **(D' and D'')**, indicating the endogenous stripe pattern of Dyl was disrupted. Scale bars: 5 μm.

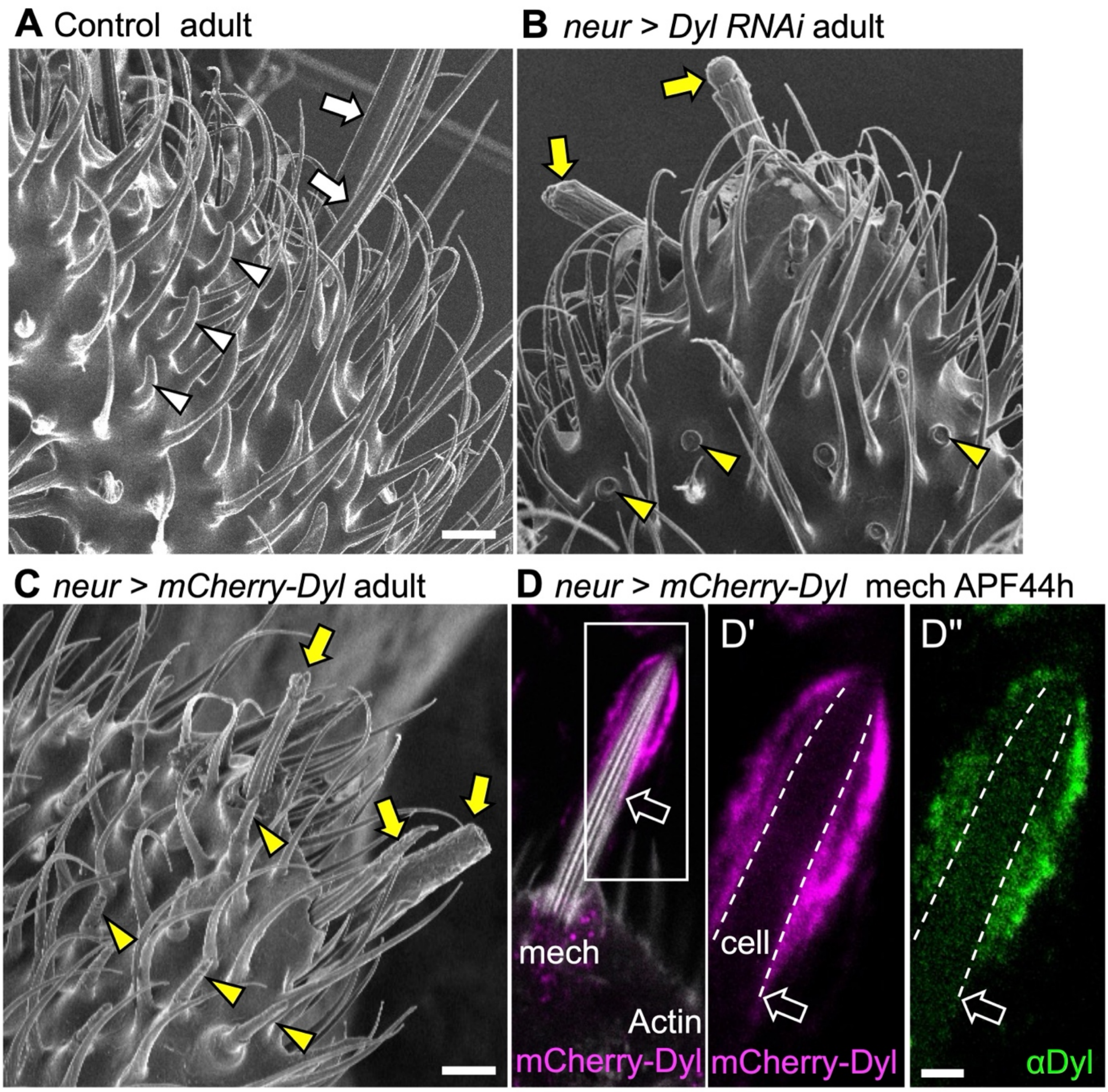
A Control adult
B neur > Dyl RNAi adult
C neur > mCherry-Dyl adult
D neur > mCherry-Dyl mech APF44h
mech
Actin
mCherry-Dyl
D'
cell
mCherry-Dyl
D''
αDyl

Fig. S4. Supporting evidence for ECM-cell mechanical interactions in olfactory bristles. (A) Despite the RNAi-mediated suppression of Tyn, cloud ECM was observed by TEM (arrowheads: outer boundary of cloud ECM). **(B)** anti-Mey, anti-Nyo, and anti-Neo signals are present in the layer II and III cloud ECM in the absence of Tyn. **(C)** The median hair size value (width and length) of 2 to 5 hairs from each fly was plotted. Tyn-RNAi-1, induced the increased length and width of olfactory protrusions (Kruskal-Wallis tests with Dunn's post hoc and Bonferroni correction). The second RNAi line Tyn-RNAi-2 caused more variable size difference and its box plot is not displayed. Control: n=13, Tyn RNAi-1: n=7, Tyn RNAi-2: n=6 flies. **(D)** The length increase of spinules and olfactory hairs from 40 to 75 h APF. Each data point represents a median value of 2 to 5 hairs from each fly. The cells elongated rapidly at 40-48 h. **(E)** Western blot analysis of Tyn, Mey, and Nyo expressed in S2 cells with or without Np. **(F)** Tyn-actin and Mey-actin distances were measured from confocal microscopy images and presented as ECM-Cell distances. Np overexpression (Np-OE) caused significant increase of Tyn-actin distance. Increase of Mey-actin distance also appears to have increased in the Np-OE group, but statistical significance was not verified (Control: n=6 hairs from 2 flies, Np-OE: n=8 hairs from 3 flies). Mann-Whitney U test. **(G)** Np overexpression caused the change in the olf hair cell. The width (horizontal axis) but not the length (vertical axis) was increased by Np overexpression. Control: n=7, Np-OE: n=6 flies, the median values of 3 to 5 hairs from each fly, Mann-Whitney U test. **(H)** Np overexpression caused abnormality in olf (arrowheads, an enlarged hair shown in Fig. 5I), while no obvious defect was observed in mech (arrows). **(I)** TEM view of the control mech and the socket (black arrowheads). The plasma membrane (PM) of the region that is free of actin bundles is dechorated with membrane protrusions **(I')**, while PM adjacent to the actin bundle was flat **(I'')**. In each case, the envelope was straight (arrows). **(J)** Spinules, rich in actin bundles, showed straight PM and envelope. Arrowhead: PM, arrow: env. Scale bars: 5 µm for **(B)**, 1 µm for **(A)**, **(H)**, and **(I)**, 100 nm for **(I')**, **(I'')**, and **(J)**. Protrusion length and width in **(C)**, **(D)** and **(G)** were measured from confocal images, using only those protrusions oriented parallel to the Z-plane.

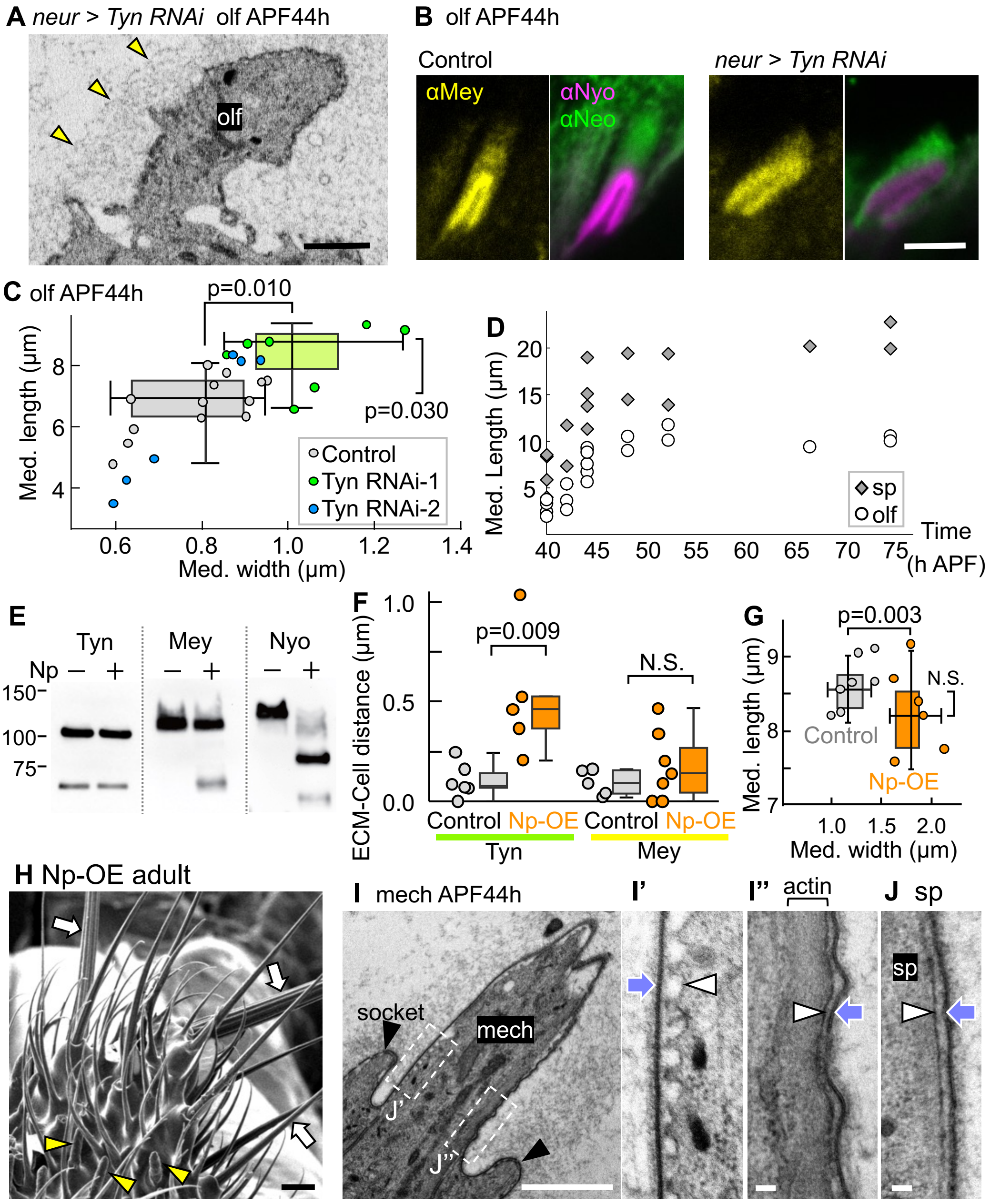
A neur > Tyn RNAi olf APF44h
olf
B olf APF44h
Control
neur > Tyn RNAi
αMey
αNyo
αNeo
C olf APF44h
p=0.010
p=0.030
Med. length (µm)
Med. width (µm)
Control
Tyn RNAi-1
Tyn RNAi-2
D
Med. Length (µm)
sp
olf
Time (h APF)
E
Tyn
Mey
Nyo
Np
150
100
75
F
ECM-Cell distance (µm)
p=0.009
N.S.
Control
Np-OE
Tyn
Mey
G
p=0.003
N.S.
Med. length (µm)
Med. width (µm)
Control
Np-OE
H Np-OE adult
I mech APF44h
socket
mech
J'
J''
I'
I'' actin
J sp
sp

Movie S1. (separate file) Constraint of cell shape and movement by expressed Tyn ECM. Note that a part of the cell covered by Tyn is constricted. S2 cells carrying the Myc::Tyn construct under the control of the metallothionein promoter were induced by the addition of 500 µM CuSO4. The recording started 160 min after induction. Tyn expression was detected with Alexa 488-labeled anti-Myc antibody added to the culture medium.

Movie S2. (separate file) Dyl ECM formation and its splitting by cell division. Myc::Dyl cells were induced and recorded as in Movie S1. Cell division: arrows at 790-800 min.

Movie S3. (separate file) Expansion and rupture of Tyn ECM. Myc::Tyn cells were induced by 500 µM CuSO4 for 2 days and were imaged with the addition of Alexa 488-labeled anti-Myc antibody. As the cells grew, Tyn ECM was stretched and broken (90 min, arrowheads), and shrunk. Correspond to Fig. S2M.

Data S1. (separate file) Reagent and strain list. This Excel file consists of five sheets, each containing a list of “Antibody”, “Plasmid”, “Reagent”, “Experimental models” and “Hardware and software”.
